# Evidences Suggesting Involvement of Viruses in Oral Squamous Cell Carcinoma

**DOI:** 10.1155/2013/642496

**Published:** 2013-12-19

**Authors:** Kanupriya Gupta, Rashmi Metgud

**Affiliations:** Department of Oral and Maxillofacial Pathology, Pacific Dental College and Hospital, Udaipur, Rajasthan 313001, India

## Abstract

Oral cancer is one of the most common cancers and it constitutes a major health problem particularly in developing countries. Oral squamous cell carcinoma (OSCC) represents the most frequent of all oral neoplasms. Several risk factors have been well characterized to be associated with OSCC with substantial evidences. The etiology of OSCC is complex and involves many factors. The most clearly defined potential factors are smoking and alcohol, which substantially increase the risk of OSCC. However, despite this clear association, a substantial proportion of patients develop OSCC without exposure to them, emphasizing the role of other risk factors such as genetic susceptibility and oncogenic viruses. Some viruses are strongly associated with OSCC while the association of others is less frequent and may depend on cofactors for their carcinogenic effects. Therefore, the exact role of viruses must be evaluated with care in order to improve the diagnosis and treatment of OSCC. Although a viral association within a subset of OSCC has been shown, the molecular and histopathological characteristics of these tumors have yet to be clearly defined.

## 1. Introduction

The significant role of viruses in cancer was acknowledged finally in the second half of the past century after various rodent tumorigenic viruses were discovered, and evidence had accumulated supporting an association between viruses and human cancer. Indeed, the Nobel Prize was awarded to Rous in 1966 in recognition of his seminal discovery of tumor-inducing viruses. In addition, almost at the same time, a Special Virus Cancer Program (VCP) was launched by the US Congress in 1964 providing enormous funds for intensive research into the supposed role of viruses in human cancer. This program, criticized by some investigators as being a political moonshot-style plan, failed to identify candidate human cancer-causing viruses yet generated fundamental information about the molecular biology and mechanisms underlying, in particular, virus-related animal cancer and cancer in general [1].

The strong cohort effect that accounted for the increased incidence of head and neck cancers after 1915 indicates that oral cancer is a disease largely attributable to behaviors that expose an individual to environmental carcinogens. The majority of oral cancers in individuals above and below the age of 45 can be attributed to the combined effects of alcohol and tobacco smoking. Other risk factors for oral cancers include diet, Body Mass Index, oral hygiene, and viral infections [2]. The most commonly implicated viruses in oral cancer transformation have been the human papillomavirus (HPV) [3, 4], herpes group viruses [5], adenoviruses [6], and the hepatitis C viruses [7, 8].

Of these, HPV and herpes have been the most thoroughly studied and are now considered to be the most likely “synergistic viruses” involved in human oral cancer. The herpes viruses most often linked to oral cancer are the Epstein-Barr virus (EBV), human herpes virus- (HHV-) 8, and cytomegalovirus (CMV) [9].

This review will attempt to focus on the approaches taken to discover human cancer viruses and promising methods for detecting new viruses. We also discuss how causal association is established and possible cofactors that influence development of virus-associated cancers.

## 2. Criteria for Defining Viral Carcinogenesis 

Even though human oncogenic viruses belong to different virus families and utilize diverse strategies to contribute to cancer development, they share many common features. One key feature is their ability to infect but not kill their host cell. In contrast to many other viruses that cause disease, oncogenic viruses have the tendency to establish long-term persistent infections [10]. Consequently, they have evolved strategies for evading the host immune response, which would otherwise clear the virus during these persistent infections. Additional cofactors, such as host immunity and chronic inflammation, as well as additional host cellular mutations, also play an important role in the transformation process [11]. Different guidelines have been proposed to aid in establishing a causal relationship between viruses and human cancers [12–15].

Evans and Mueller Guidelines [13].

*Epidemiologic Guidelines*

1. Geographic distribution of viral infection corresponds with that of tumor, adjusting for the presence of known cofactors.
2. Viral markers are higher in case subjects than in matched control subjects.
3. Viral markers precede tumor development, with a higher incidence of tumors in persons with markers than those without.
4. Tumor incidence is decreased by viral infection prevention.

*Virologic Guidelines*

1. Virus can transform cells *in vitro*.
2. Viral genome is present in tumor cells but not in normal cells.
3. Virus induces the tumor in an experimental animal.

*Hill Criteria for Causality [14, 15]*

1. Strength of association (how often is the virus associated with the tumor?).
2. Consistency (has the association been observed repeatedly?).
3. Specificity of association (is the virus uniquely associated with the tumor?).
4. Temporal relationship (does virus infection precede tumorigenesis?).
5. Biologic gradient (is there a dose response with viral load?).
6. Biologic plausibility (is it biologically plausible that the virus could cause the tumor?).
7. Coherence (does the association make sense with what is known about the tumor?).
8. Experimental evidence (is there supporting laboratory data?).

## 3. Human Papillomavirus and OSCC

The human papillomavirus (HPV) family consists of more than 200 genotypes, classified in accordance with the ability to infect and transform epithelial cells. HPVs are DNA viruses that specifically target the basal cells of the epithelial mucosa [16]. Genotypes, such as HPV1, infect epidermal cells, whereas HPVs 6, 11, 16, and 18 infect epithelial cells of the oral cavity and other mucosal surfaces [17].

HPV belongs to the family Papovaviridae. These are small nonenveloped icosahedral viruses with an 8 kbp long double-stranded circular DNA genome. The papilloma virus genome comprises early and late genes that encode early proteins E1–E7 and late proteins L1-L2. The early proteins are nonstructural proteins involved in replication and transcription of the genome (E1–E5) or in host cell tumoral transformation (E6 and E7), whereas L1 and L2 are the structural capsid proteins of the virion. The late genes (L1-L2) code for the structural proteins of viral capsid and are activated during the final stages of the viral cycle. Up to six early genes and two late genes can be detected in HPV [18–23] (Table 1).

The ability of HPV to transform epithelial cells is divided into high-risk and low-risk types. Low-risk types are associated with development of benign lesions such as warts, while infections with high-risk types may progress to malignant lesions [17].

Both high-risk and low-risk types of HPV can cause the growth of abnormal cells, but only the high-risk types lead to cancer because only the E7 protein encoded by high-risk HPVs can immortalize human epithelial cells. Sexually transmitted, high-risk HPVs include types 16, 18, 31, 33, 35, 39, 45, 51, 52, 56, 58, 59, 66, 68, and 73 [24]. It is important to note, however, that in the genital tract the large majority of high-risk HPV infections regress on their own and do not cause cancer [25].

The HPV involvement in oral carcinogenesis was supported on the basis of the following evidences:

1. the strongly established etiological role of HPV in cervical SCC [26, 27];
2. the epithelial tropism of HPV;
3. the similarity between oral and genital epithelia [28];
4. the detection of HPV genotypes in samples of OSCC [29–33].

## 4. Genital HPV Infection and Risk of OSCC

Several studies examined the incidence of second cancers after an initial diagnosis of anogenital cancers [34, 35] and have showed that there is an increased risk of head and neck cancer as well as other HPV-associated anogenital cancers in the two major HPV 16 oncogenes E6- and E7-positive patients. This association between HPV-associated anogenital cancers and head and neck cancer was further strengthened by two larger studies [36, 37]. Additionally, the presence of antibodies to HPV E6 and E7 proteins was found to be more associated with tumors of the oropharynx than with these of the oral cavity.

A recent study has shown that besides the classical horizontal transmission during the sexual life, a vertical transmission occurs in approximately 20% of case HPV-positive people. In these individuals, HPV-DNA is detected in amniotic fluid, foetal membranes, blood, and placental trophoblastic cells, all suggesting HPV infection in utero, that is, prenatal transmission [38].

### 4.1. Human Papillomavirus- (HPV-) Induced Carcinogenesis

E6 and E7 oncoproteins can inactivate the genetic mechanisms that control both the cell cycle and apoptosis. The hallmark of high-risk HPV E6 oncogenic activity is degradation of the p53 tumour-suppressor gene [39, 40]. The functions of p53 in the cell cycle include controlling the G1 transition to the S phase of the cell cycle at the G1 checkpoint by inducing expression of cyclin inhibitors p16, p21, and p27 that block the activities of cyclin-CDKs (cyclin-dependent kinases) complexes, thus mediating arrest of the cell cycle by blocking the progression of the cell cycle at the G1/S transition. p53 activities mediate cell proliferation in response to mitogenic stimulation; mediate arrest of the cell cycle growth at the G1 checkpoint following DNA damage, hence permitting repair of the damaged DNA before the cell enters the DNA synthesis phase; and mediate induction of apoptosis of cells in which the DNA damage is beyond repair. Therefore, inactivation, degradation, or mutation of the p53 gene may dysregulate its functions resulting in increased cell proliferation, in accumulation of damaged DNA, in growth of cells harbouring DNA errors, and in prolonged cell survival. However, loss of p53 function alone is not sufficient for the development of cancer, and other cytogenetic alterations are required for complete malignant transformation [41].

In addition to these properties, E6 oncoprotein of high-risk HPV types can also mediate cell proliferation through the PDZ-ligand domain. The PDZ domain is located at areas of cell-to-cell contact, such as tight junctions of epithelial cells, and is associated with signal transduction pathways. The binding of high-risk HPV E6 oncoprotein to the PDZ family of proteins may result in degradation of the PDZ domain leading to dysregulation of organization, differentiation, and the chromosomal integrity of HPV infected epithelial cells [41]. This may contribute to morphological transformation of keratinocytes infected with high-risk HPV and to induction of epithelial hyperplasia. Telomerase is an enzyme that adds hexanucleotide repeats onto the end of the chromosome telomere. Telomerase activity is usually restricted to embryonic cells and is absent in normal somatic cells. When telomerase is absent, there is progressive shortening of telomeres as the cells repetitively divide, ultimately resulting in senescence of these cells [42].

HPV-induced activation of telomerase prevents the shortening of telomeres resulting in prolongation of the life-span of HPV-infected cells. High-risk HPV E7 oncoprotein has the capacity to initiate DNA synthesis in differentiated epithelial cells mainly by binding and inactivating the Rb apoptosis/tumour suppressor gene. The Rb family of proteins plays an essential role in controlling the cell cycle by governing the checkpoint between the G1 and the S phase. Hypophosphorylated Rb binds to E2F transcription factor forming a Rb-E2F complex, making E2F unavailable for transcription of genes associated with DNA synthesis. Upon phosphorylation of Rb by cyclin-CDK complexes, E2F is released from the Rb-E2F transcription repressor complex, and it induces transcription of the S-phase genes. E7 oncoprotein of high-risk HPV types functionally inactivates the Rb family of proteins resulting in overexpression of E2F transcription factor with upregulation of cell cycle genes resulting in DNA replication, in the transition of the cell from the G1 to the S phase, and in increased cell proliferation [43–45].

E7 oncoprotein can also interact with other cellular factors that control the cell cycle including histone deacetylases, AP-1 transcription complex, and CDK inhibitors, p21 and p27. Furthermore, E7 of high-risk HPV 16 and HPV 18 can decrease the expression of major histocompatibility complex (MHC) class I molecules, thus interfering with MHC class I antigen presentation, resulting in downregulation of cellular immune responses, allowing HPV to persist in infected epithelial cells [46].

In addition to these properties, high-risk HPV E7 oncoprotein can induce chromosome duplication errors leading to dysregulation of mitotic spindle formation and function, contributing to the genomic instability of the cell. The separate pathological effects of high-risk HPV E6 and E7 on the cell cycle complement each other, and together E6 and E7 mediate the HPV-associated epithelial cell transformation and promote cellular genomic instability that predisposes the infected cells to full malignant transformation. High-risk HPV E7 activates the DNA synthesis and cell replication mechanisms that are normally inactive in matured epithelial cells, thus initiating pathological cell growth. By inducing cell survival and delayed apoptosis of cells with DNA damage, E6 allows E7 to exert and sustain its pathological effect [47].

**Figure 1:**
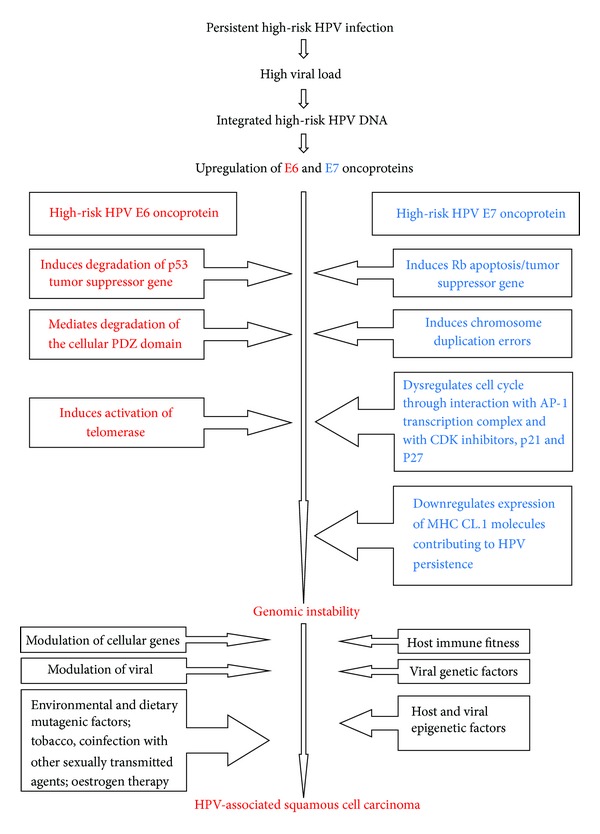


Typically, infected epithelial cells of HPV-associated benign lesions harbor low-risk HPV episomally in the nuclei. In HPV-associated malignancies, high-risk HPV DNA may either be integrated within the cellular genome or it may be maintained as an episome in the nuclei of the malignant cells. It is unclear how the HPV genome, whether episomal within the nucleus or integrated into the nuclear cellular genome, brings about the same end result of malignancy. The integration of HPV DNA favours the inactivation of tumour suppressor genes, p53 and Rb, contributing to increased cellular chromosomal instability and prolonging the life-span of the cell, which are essential steps in the multistep process of HPV-associated carcinogenesis. It is probable that following the initial HPV-induced cellular transformation, additional interactions with chemical carcinogens will provide the necessary additional impetus for the development of frank malignancy [47] (Figure 1).

The HPV involvement in oral and oropharyngeal carcinogenesis was first proposed in 1983 by Syrjänen et al. [121] (Table 2). Their results showed that 40% of the laryngeal and oral cancers contained histological and morphological similarities with HPV-infected lesions, and 50% of the samples demonstrated HPV structural proteins by immunohistochemistry [122]. Since then, several studies have focused on HPV detection in oral cancer but results have been conflicting [65, 123, 124]. It was found that the prevalence of HPV detection varies broadly, depending on the population, on the location of the cancerous lesion, on type of specimen, and on detection method. By contrast, HPV was more frequently detected in OSCCs of the oropharynx and tonsil than at other head and neck sites [125–127]. However, in a systematic review that was performed by Syrjänen et al. [128] it was suggested that a potentially important causal association between HPV (specifically HPV 16) and OSCC exists. In a recent study the overall HPV prevalence was 10.5% in oral cavity carcinomas and was higher in female than in male cases. Ninety-five percent of HPV-positive cases were infected by a single HPV type. HPV 16 was the most prevalent type and was found in 95.5% of HPV-positive oral cavity carcinoma cases [129].

Furthermore, other studies have proved the existence of a synergistic effect between HPV and alcohol. The risk of head and neck cancer was statistically significantly increased in heavy alcohol users detected with the virus, compared to that of HPV-negative cancer drinkers. Therefore, it has been proposed that alcohol can biologically modify mucosal tissue, by increasing its permeability to viral infection or by influencing the immune response to HPV [99]. It is believed that one of the major events of HPV-induced carcinogenesis is the integration of the HPV genome into a host chromosome. HPV genome integration often occurs near fragile sites of the human genome, but there are no apparent sites for integration and no evidence for insertional mutagenesis [130].

Slightly modifying Koch's postulates, in order to establish a relationship between a causative virus and a disease, four criteria are needed: (1) viral genome ought to be present in tumor lesions or in tumor cells; (2) virus must be isolated from a pathologic lesion and grown in culture; (3) cultured virus should cause disease when inoculated into a healthy organism; and (4) virus must be reisolated from the inoculated host and identified as being identical to the original specific causative factor. However, the use of Koch's postulates to establish disease causation does not fully apply to these phenomena, since the etiology of cancer is multifactorial [131]. On the contrary, other authors suggest that the incidence of HPV infection in the oral cavities of healthy population is very low and therefore other risk factors are most likely responsible to promote oral carcinogenesis [132]. Hence, we have tried to collect the studies done on HPV in oral cancer.

## 5. HPV Testing

HPV testing is critical for the estimation of HPV prevalence in various oral diseases. HPV testing is usually based on PCR method. General or consensus primers targeting L1 gene are most frequently used for HPV detection because they are able to identify several HPV genotypes at the same time. Sampling techniques together with widely divergent PCR methods in different studies explain most of the variability in HPV prevalence among OSCC and control samples [133]. *In situ* hybridization and *in situ* oncogenic protein staining techniques have also increased sensitivity and specificity and are used for HPV testing. These techniques have allowed for not only the detection of HPV in cytological smears or histopathological immunesections but also the determination of the topographic site of the infection [134]. According to recent studies, HPV-positive squamous cell carcinomas have intact p16 gene and wild type p53 compared to HPV-negative ones [135]. Other authors have noted that a distinctive mark of the presence of HPV in oral cancer could be found in p16 nuclear or cytoplasmic overexpression [136, 137]. However, one goal of the scientific research is to find new biological markers able to identify the set(s) of genes involved in oral carcinogenesis.

## 6. HPV Serology

The immune response to HPV infection involves both the cell-mediated and humoral responses. However, serological evidence is circumstantial since it provides only data on prior exposure to HPV. Since not all patients with HPV-associated cancers have detectable HPV antibodies, serum antibody determination may be a limited biomarker for HPV infection and carcinogenesis. Serum antibodies to HPV capsid proteins (virus-like particles) are thought to be a marker of lifetime HPV infection [138, 139]. Antibodies against HPV E6 and E7 proteins are associated with increased risk of HPV-associated cancer [140, 141] but are rather linked more with tumors from the oropharynx than from the oral cavity. The use of HPV viral load in oral biopsies in conjunction with serological markers may serve to identify a subset of HPV-associated oral cancers in which HPV is biologically active.

## 7. Herpes Simplex Virus (HSV)

HSV is a double-stranded DNA virus that is enveloped. When it infects cells the series of events that takes place is very complex but can be analyzed logically since it follows a coordinated series of steps. The virus exists in two closely related forms, known as HSV 1 and HSV 2. HSV 1 causes mainly oral and ocular infections, while HSV 2 causes mainly genital infections. However, the overall behavior and structure of the two viruses are very similar. Each virus causes a severe primary infection followed by a latent infection that may be followed by recurrent infections. Cells that are infected by HSV are killed. Gene maps of HSV 1 and HSV 2 have been developed and show that the various functions of the two viruses are encoded by identical regions of the genome of each virus [142].

The only viral function that is not in a related location between the two types is the ability to transform cells, and the reason is unknown. The transforming region of HSV 1 (minimum transforming region 1, mtr-1) is located in the left third of the genome, while the equivalent two functions of HSV 2 (mtr-2 and mtr-3) are close to the center of the genome [143].

### 7.1. Transformation by HSV

Induction of cellular proteins has been studied as a possible mechanism for transformation by HSV. It is known that infection by HSV 1 induces the expression of “stress” or “heat shock” proteins. The mechanism of induction is not known but does depend on the expression of the immediate-early family of HSV proteins. Since HSV might transform cells by stimulating the expression of cellular proteins, some workers have started to study such proteins by isolating cDNA from cells that were transformed by HSV 2 [142–144].

However, the function of these cloned fragments is still not known. Host cell shutoff is recognized as being an important event during infection of cells by HSV. The infected cell ceases to synthesize cellular proteins, and cell RNA is degraded very quickly. Recently, it was found that the gene of HSV that mediates shutoff is located in the same region of the genome as the mtr-2 region of HSV 2, which mediates cell transformation [145]. This has raised the possibility that the mechanism of transformation might be related to the mechanism of shutoff. Shutoff is known to be a multistep process, depending on more than one viral activity [146].

The first phase of host cell shutoff is due to some activity by the virus particle itself. It happens very soon after infection and occurs even if virus gene expression is prevented by Actinomycin D or by irradiation of the virus [147].

The second phase of the host cell shutoff does require the expression of virus genes and eliminates any remaining host protein synthesis. It seems likely that these phenomena will be studied quite closely in the future to find which of them is most likely to be involved in cell transformation. Other activities of HSV that might be related to cell transformation include the fact that the virus can stimulate the replication of other viruses. This can also occur when cells are exposed to chemical carcinogens [148].

#### 7.1.1. Induction of Cellular Proteins

Infection by HSV induces the expression of stress or heat shock proteins. According to Steele and Shillitoe, the exact mechanism is not known but it depends on the expression of the immediate early family of HSV proteins. It was, therefore, concluded that HSV might transform cells by stimulating the expression of cellular proteins [149].

#### 7.1.2. Host Cell Shutoff Process

Infected cell ceases to synthesize cellular proteins and cell RNA would be very quickly degraded. A mediating gene was located in the same region of the genome as the mtr-2 region of HSV 2 that mediates cell transformation [145]. Steele and Shillitoe raised the possibility that the mechanism of cell transformation might be related to the mechanism of shutoff [149].

#### 7.1.3. Stimulation of Other Viruses by HSV

This comes from the idea that at least in cervical carcinogenesis, HSV and HPV may act as cocarcinogens, with HSV as an initiator and HPV as a promoter. In oral lesions, however, Scully et al. in 1993 showed that very few premalignant or carcinoma specimens appeared to have both HPV 16 and HSV 1 DNA sequences. They, therefore, concluded that there is no evidence that these potentially oncogenic DNA viruses do play a synergistic role in oral cancer development, but the possibility cannot be discounted [150].

#### 7.1.4. Chromosomes as Targets

Stich et al. [151], Mincheva et al. [152], and Peat and Stanley [153] suggested that when cells are infected by HSV there is chromosomal damage which is at first restricted to a site on chromosome 1q and to some extent on chromosomes 3, 9, and 16. Based on these studies, Steele and Shillitoe gave a plausible explanation for the possibility that HSV has specific chromosomal targets for rearrangement. Damage to a particular chromosomal site might be another possible mechanism of cell transformation by HSV [149].

El Sissy [154] attempted to search for possible presence of HSY-2 protein in 21 lesional tissues of oral squamous cell carcinoma, as well as in normal oral mucosa, using immunohistochemical peroxidase-antiperoxidase (PAP) technique. Specimens of normal oral mucosa revealed frequent positive staining for polyclonal HSY-2 protein marker, thus indicating that HSY-2 is not an uncommon inhabitant of the oral mucosa. Only highly differentiated grades of oral squamous cell carcinoma showed positive staining for HSY-2, while less differentiated carcinomas failed to reveal any immunoreactivity. This raised the suggestion of a possible causative role played by HSY-2 in the establishment of the neoplastic process in oral squamous cell carcinoma through a “hit and run” mechanism. The present results point to HSY-2 as a promoter or an initiator in early neoplastic changes in well-differentiated oral squamous cell carcinoma that becomes denatured and consequently not evident in less highly differentiated tumours.

Jalouli et al. [155], using PCR/DNA sequencing, investigated the prevalence of human papillomavirus (HPV), herpes simplex virus (HSV), and Epstein-Barr virus (EBV) DNA in brush biopsies obtained from 150 users of Sudanese snuff (toombak) and 25 nonusers of toombak in formalin-fixed paraffin-embedded tissue samples obtained from 31 patients with oral dysplasias (25 toombak users and 6 nonusers) and from 217 patients with oral cancers (145 toombak users and 72 nonusers). In the brush tissue samples from toombak users, HPV was detected in 60 (40%), HSV in 44 (29%), and EBV in 97 (65%) of the samples. The corresponding figures for the 25 samples from non-users were 17 (68%) positive for HPV, 6 (24%) positive for HSV and 21 (84%) for EBV. The formalin-fixed samples with oral dysplasias were all negative for HPV. In the 145 oral cancer samples from toombak users, HPV was detected in 39 (27%), HSV in 15 (10%), and EBV in 53 (37%) of the samples. The corresponding figures for the samples from nonusers were 15 (21%) positive for HPV, 5 (7%) for HSV and 16 (22%) for EBV. These findings illustrate that prevalence of HSV, HPV, and EBV infections is common and may influence oral health and cancer development.

Delavarian et al. [156] investigated the presence of viruses in oral squamous cell carcinoma (OSCC) in young patients (20–40 years old) attending Mashhad Dental Faculty from 1996 to 2009 for the first time in Iranian population. Twenty-one formalin-fixed, paraffin-embedded sections of patients under 40 years with clinical diagnosis of OSCC, who had been referred to Mashhad Dental Faculty from 1996 and 2009, were evaluated for DNA extraction. All specimens were tested for presence of human papillomavirus, Epstein-Barr virus, Herpes simplex virus type 1, and cytomegalovirus virus. From 21 specimens, viruses were detected only in three cases. Two samples were positive for EBV and the third one was coinfected with EBV and HSV 1. All specimens were negative for HPV and CMV. Authors concluded that viruses had no important role in OSCC in young patients. Further researches are needed to clarify this role and to identify other possible risk factors.

#### 7.1.5. Epidemiological Studies

The herpes simplex viruses (HSVs), types 1 and 2, have been investigated in the past for possible associations with human cancers. Levels of antibody to HSV 1 have been reported to be higher in patients with oral cancer than in control subjects [157]. These antibodies were largely of the IgA and IgM classes, and patients with the highest levels of anti-HSV IgM had a shorter survival than other patients [158]. It has been reported that a combination of HSV seropositivity and a history of cigarette smoking is associated with a higher risk of oral cancer than would be expected from a purely additive effect [159]. Early studies attempted to find if DNA of HSV could be detected in oral cancers, and preliminary reports did indicate the presence of both viral DNA and RNA [58, 160]. However, the nature of the HSV genome makes it difficult to produce specific probes, and the possible sequences that were detected have not been identified [58].

### 7.2. Molecular Mechanisms of Carcinogenesis

The explanation for the association between oral cancer and HSV could well be that of a confounding variable, except for the fact that HSV can transform some animal cells to a malignant phenotype *in vitro* [161]. Indeed HSV has shown cocarcinogenic activity in combination with chemicals *in vivo* [162]. Unfortunately, the association is difficult to study, because cells that are transformed by HSV do not express specific virus antigens or retain any specific genes of the virus [163]. Instead it seems likely that the transformation is due to the virus acting as a mutagen, and a region of the viral genome has been isolated which raises the mutation frequency in cultured cells [164]. This results in some but not all of the features of malignancy [165]. Neither the mutations nor the phenotypic changes are sufficiently specific to act as markers by which a herpes-induced malignancy could be diagnosed [166]. Recent years have seen little or no progress in the study of HSV and its malignant potential.

## 8. Epstein-Barr Virus Infection (EBV)

EBV was named after Tony Epstein and Yvonne Barr who were the two scientists who first isolated and described the virus in 1964 from lymphoma samples collected by Denis Burkitt. The EBV genome is a double-stranded DNA molecular of approximately 172,000 base pairs; as in other herpes viruses, the molecule is divided into unique, internal repeat, and terminal repeat domains [167]. The genome encodes approximately 80 proteins. The function of many of the genes involved in viral replication has been inferred from their homology to herpes simplex virus genes; however, genes expressed during latent infection of B cells do not have recognized counterparts in other human herpes viruses.

All phases of the EBV life cycle are associated with human disease. In immunocompromised individuals, infected cells increase in number and eventually B cell growth control pathways are activated, inducing transformation and leading to malignancies such as nasopharyngeal carcinoma (NPC), Burkitt's lymphoma (BL), posttransplant lymphomas, and gastric carcinomas [168]. EBV encodes several viral proteins that have transforming potential, including EBV latent membrane proteins 1 and 2 (LMP1 and LMP2) and EBV nuclear antigens 2 and 3 (EBNA2 and EBNA3). LMP1 can transform a variety of cell types, including rodent fibroblasts [169], and is essential for the ability of EBV to immortalize B cells [170]. The multiple transmembrane-spanning domains and the carboxyl terminus of LMP1 can interact with several tumor necrosis factor receptor associated factors (TRAFs) [171, 172]; this interaction results in high levels of activity of NF-jB, Jun, and p38 in LMP1-expressing epithelial and B cells [173–175].

LMP1 also upregulates the expression of numerous antiapoptotic and adhesion genes and activates the expression of IRF-7 [176], matrix metalloproteinase-9 (MMP-9), and fibroblast growth factor-2 (FGF-2) [177]. A second viral membrane protein, LMP2, is dispensable for transformation of naïve B cells but is required for transformation of postgerminal center B cells. LMP2 interacts with Lyn and Syk to mimic B cell receptor (BCR) signaling, including activation of the PI3 K/AKT survival pathway [178]. These pathways are constitutively active and drive proliferation through a normal cell cycle cascade.

Horiuchi et al. [179] carried out a study to determine the presence of EBV in various squamous cell proliferative lesions in the oral cavity. They made use of PCR and *in situ* hybridization for detecting the presence of EBV DNA and EBV encoded small messenger RNA. Nearly 60% of SCC was EBV genome positive, but none of papilloma demonstrated EBV genome. On the other hand, oral hairy leukoplakia lesion seen in patients with AIDS has been proved to be EBV associated. Horiuchi et al. concluded that EBV virus infection of oral squamous epithelium may be carcinogenic or, alternatively, the virus may merely exist in epithelial cells of squamous cell carcinoma, carcinoma *in situ,* and leukoplakia.

González-Moles et al. [180] showed a positive correlation between different grades of OSCC and EBV DNA positivity and also showed that percentage positivity of EBV DNA increases from well-differentiated OSCC to poorly differentiated OSCC. In a study by Higa et al. [181], fifty-four patients with oral squamous cell carcinoma reported from 1997 to 1999 in Okinawa were compared with 21 and 20 patients from Kitakyushu and Kumamoto in Kyushu, mainland Japan, respectively. 51 diagnoses were confirmed by conventional histological examination of paraffin wax sections. EBV was detected by nonisotopic *in situ* hybridization and PCR (Bam HI-F, EBV nuclear antigen 2 (EBNA2), and latent membrane protein 1 (LMP-1) regions). Sequence analysis of the PCR products was also carried out. In Okinawa, 25 patients were found to be infected with EBV type A by analyzing the 3-sequence divergence of the EBNA2 genes. Six patients were positive for EBV type B and 8 for both types A and B. Therefore, type A virus infection was demonstrated in 33 of 54 patients and type B in 14 of 54. In total, 39 of 54 patients were infected with EBV. Authors concluded that, in Okinawa, EBV infection was frequently demonstrated in oral squamous cell carcinoma (*P* < 0.001). However, in mainland Japan, there was no significant correlation between EBV and oral squamous cell carcinoma [181].

A study by Li et al. [182] to detect EBV infection and gene expression in oral cancer from patients in Taiwan by microarray analysis revealed that the majority of the specimens (82.5%) were EBV positive that probably expressed coincidently the genes for EBNAs, LMP2A, and 2B and certain structural proteins. Importantly, the genes fabricated at the spots 61 (BBRF1, BBRF2, and BBRF3) and 68 (BDLF4 and BDRF1) on EBV-chip were actively expressed in a significantly greater number of OSCC exhibiting exophytic morphology or ulceration than those tissues with deep invasive lesions.

In a study conducted by Sand et al. [183] examining 29 patients with OSCC, 23 with OLP, and 67 with clinically healthy oral mucosa, a nested polymerase chain reaction method for EBV DNA analysis was used. The overall EBV prevalence in patients with oral disease was 32.1%. Of the OSCC patients, 37.9% were EBV positive, and, of the OLP patients, 26.1% were EBV positive. Both percentages were statistically significant compared with that of control patients (7.3%). The difference in EBV prevalence between the smoking control group and the nonsmoking control group was without significance. Increased age did not enhance EBV prevalence. The authors were of the opinion that EBV is present in oral diseases such as OSCC and OLP. Smoking, alcohol use, or age does not seem to be a risk factor for EBV infection.

Jiang et al. [184] isolated epithelial cells by laser capture microdissection and levels of CD21, CK19, and EBV RNA were measured by quantitative reverse transcriptase PCR in oral dysplasia and squamous cell carcinoma. Results showed that expression of CD21 increased in frequency and intensity as oral epithelial cells become more dysplastic and that expression correlates with an increase in infection by EBV. Tumors or dysplastic lesions that carry EBV also generally express higher levels of CK19 than those that do not. Hence, the findings suggest that dysplasia may make cells more susceptible to infection by EBV and that infection by the virus may alter the phenotype of the infected cell in a manner which could affect prognosis.

## 9. Hepatitis C Virus

The worldwide prevalence of hepatitis C virus (HCV) is estimated to be around 3%, representing approximately 170 million infected individuals. Morbidity associated with HCV infection is due to not only the sequelae of chronic liver disease but also a variety of extra hepatic manifestations, including that involving the oral cavity [185].

Hepatitis C virus (HCV) is an enveloped, single-stranded positive-sense RNA virus that was isolated in 1989 from a chimpanzee chronically infected by contamination with a human factor VIII concentrate (Choo et al.) [186].

There are six HCV genotypes and more than 100 subtypes. Its envelope contains two glycoproteins, E1 and E2, which form heterodimers at the surface of the virion. The genomic RNA is translated into a viral polyprotein which is cleaved by cellular proteases to generate the capsid protein (C), the two glycoproteins E1 and E2, a small protein the role of which is unclear (p7), viral proteases NS2 and NS3, and nonstructural proteins NS4A and 4B and NS5A and 5B, which are required for viral RNA replication [187].

Although details of HCV replication are not known, it is thought to take place in the cytoplasm, where, among other products, negative-sense viral RNA (vRNA), replicative double-stranded forms, and nonstructural proteins are synthesized. Their presence can thus be used as evidence that virus multiplication, as opposed to passive transportation, is occurring (Negro et al.) [188]. Furthermore, sequence variants forming a quasispecies may circulate within an individual, possibly as a consequence of ongoing immune surveillance and viral mutations (Toyoda et al.) [189].

Johnson et al. suggested a possible involvement of HCV in diseases outside the liver and concluded that since oral cavity is frequently exposed to HCV viruses, this in turn increases the risk of genetic instability in these cells [185]. Nagao et al. studied a group of 100 patients including 88 SCC. Anti-HCV antibodies were detected in sera of 25 patients [190]. The exact mechanism is unclear. Gandolfo et al. found very high prevalence of anti-HCV antibodies in patients with oral lichen planus (OLP) [191]. Nagao et al. suggested that, since OLP is also histologically a disease of squamous cells, the squamous cells of oral region are continuously exposed to HCV from saliva as well as from serum in HCV-positive patients and that this may be involved in the development of SCC and OLP in these patients [190].

## 10. Conclusion

Oral cancer is an important cause of morbidity and mortality, especially in developing countries, and its prevalence may rise in the foreseeable future. The studies of virus-associated head and neck cancers have provided many critical insights into key mechanisms of carcinogenesis. The human tumor virus oncogenes play central roles in viral life cycles and their oncogenic potential is a manifestation of these activities.

Some viruses, most notably the high-risk HPVs, play essential roles in the initiation as well as progression of cancers and continued expression of their viral transforming activities is necessary for the maintenance of the transformed phenotype. Recent reviews establish the increased evidence of HPV-related oral cavity cancer in man.

Standardization of the methods for sample collection and analysis is mandatory to obtain reliable data and to compare the results obtained in different studies in the presence of HPV in variable proportions in oral squamous cell carcinoma tissues. Some tumors are associated with papillomaviruses and some with viruses of the herpes family; however, the exact role of these viruses must still be evaluated carefully. These viruses may provide targets for therapy and for diagnostic tests and may widen our understanding of the mechanisms by which the tumors develop.

Vaccines are designed strictly for prevention of such viral infections which may be involved in cancers of the head and neck. Few studies found sufficient evidence to support a global immunization program against HPV, irrespective of gender and geography, to help to achieve a reduction in HPV-related malignant diseases in the future. Clearly, large, prospective randomized trials are needed to document the clinical usefulness of these vaccines against oral cancer.

## Figures and Tables

**Table 1:** Overview of HPV gene products (E) early and (L) late.

Gene product	Description
E1	Helicase function; essential for viral replication and control of gene transcription.
E2	Viral transcription factor; essential for viral replication and control of gene transcription.
E4	Interaction with cytoskeleton proteins; viral assembly.
E5	Growth stimulation by interaction with growth factor receptors; downregulation surface HLA class I molecules.
E6	Cell immortalization; p-53 degradation; telomerase activation; antiapoptotic effect; induction of genomic instability.
E7	Cell immortalization; interaction with pRb and pRb-associated pocket proteins; transactivation of E2F dependent promoters; induction of genomic instability.
L1	Major capsid proteins.
L2	Minor capsid protein; role in recruiting viral genomes for encapsidation; involvement in nuclear transport of viral DNA.

**Table 2:** Studies on HPV in oral squamous cell carcinoma.

Study/year	Geographic location	Tumour site(s)	No. of cases	HPV +ve *N* (%)	Genotype(s)	DNA method
Loning et al., 1985 [48]	Germany	Oscc	6	50%	16, 11	SB
Maitland et al., 1987 [49]	United Kingdom	Oscc	15	46.7%	16	SB
Gassenmaier and Hornstein, 1988 [50]	Germany	Oscc	68	23.5%	16	ISH
Syrjänen et al., 1988 [51]	Finland	Oscc	51	11.8%	16, 18	ISH
Chang et al., 1990 [52]	Finland	Oscc	40	2.5; 27.5%	16	ISH; PCR
Greer et al., 1990 [53]	USA	Oscc	100	6%	n.d	ISH
Kashima et al., 1990 [54]	USA	Oscc	26	19.2%		SB
Young and Min et al., 1991 [55]	USA	Oscc	27	0%	6, 11, 16, 18, 31, 33, 35	PCR
Zeuss et al., 1991 [56]	Spain	Oscc	15	0%	6, 11	ISH
Holladay and Gerald, 1993 [57]	USA	Oscc	39	17.9%	16	PCR
Cox et al., 1993 [58]	UK	Oscc	8	50%	16	SB
Brandwein et al., 1994 [59]	USA	Oscc	64	25%	16	PCR
González-Moles et al., 1994 [60]	Spain	Oscc	27	37%	6, 11	ISH
Ostwald et al., 1994 [61]	Germany	Oscc	26	61.5%	16, 18	PCR
Balaram et al., 1995 [62]	India	Oscc	91	73.6%	6, 11, 16, 18	PCR
Shindoh et al., 1995 [63]	Japan	Oscc	77	31.2%	16	PCR
Van Rensburg et al., 1995 [64]	South Africa	Oscc	66	1.5%	18	ISH
Cruz et al., 1996 [65]	The Netherlands	Oscc	35	54.3%	16	PCR
Mao et al., 1996 [66]	USA	Oscc	64	31%	n.d.	PCR
Van Rensburg et al., 1996 [67]	South Africa	Oscc	146	1.4%	11, 16	PCR
Chiba et al., 1996 [68]	Japan	Oscc	38	21%	16, 18, 33	PCR
Wen et al., 1997 [69]	Japan	Oscc	45	31.1%	16, 18	PCR
Gopalakrishnan et al., 1997 [70]	USA	Oscc	10	30%	16	PCR
Ibrahim et al., 1998 [71]	Sudan	Oscc	88	0; 0%	n.d.	ISH; PCR
Koh et al., 1998 [72]	Korea	Oscc	42	52%	n.d.	PCR
Premoli-De-Percoco et al., 1998 [73]	Venezuela	Oscc	50	70%	16, 18	ISH
D'Costa et al., 1998 [74]	India	Oscc	100	15%	16, 18	PCR
Schwartz et al., 1998 [33]	USA	Oscc	193	21.2%	16	PCR
Elamin et al., 1998 [75]	UK	Oscc	28	50%	6, 16	PCR
Aggelopoulou et al., 1999 [76]	Greece	Oscc	81	49%	n.d	PCR
Pillai et al., 1999 [77]	India	Oscc	61	16.8%	16	PCR
Pintos et al., 1999 [78]	Canada	Oral cavity	29	10%	n/s	PCR
Badaracco et al., 2000 [79]	Italy	Oscc	66	36.4%	16, 6	PCR
Bouda et al., 2000 [80]	Greece	Oscc	19	94.7%	16, 18, 33	PCR
Cao et al., 2000 [81]	China	Oscc	40	72.5%	16, 18	PCR
Patima Cao et al., 2000 [82]	China	Oscc	73	74%	16, 18	PCR
Sand et al., 2000 [83]	Sweden	Oscc	24	12.5%	16, 18, 6, 11	PCR
Tsuhako et al., 2000 [84]	Japan	Oscc	83	56.9%	n.d.	PCR
Gillison et al., 2000 [30]	USA	Oscc	84	11.9%	16	PCR & ISH & SB
Bouda et al., 2000 [80]	Greece	Oscc	19	89.5%	16, 18	PCR
Premoli-De-Percoco and Ramirez 2001 [85]	Venezuela	Oscc	50	60%	16, 18	PCR
Schwartz et al., 2001 [86]	USA	Oscc	254	24.4%	16	PCR
Chen et al., 2002 [87]	Taiwan	Oscc	29	82.7%	16, 18, 6	PCR
Kojima et al., 2002 [88]	Japan	Oscc	53	66%	38	PCR
Nagpal et al., 2002 [89]	India	Oscc	110	33.6%	16, 18	PCR
Chang et al., 2003 [90]	Japan	Oscc	103	49.5%	HR	PCR
Fregonesi et al., 2003 [91]	Brazil	Oscc	46	39%	16, 18, 6, 11	ISH
Kansky et al., 2003 [92]	Slovenia	Oscc	62	95%	16, 33, 58, 11, 31, 68	PCR
Ritchie et al., 2003 [93]	USA	Oscc	141	15%	16, 33	PCR
Sugiyama et al., 2003 [94]	Japan	Oscc	86	34.8%	16	PCR
Herrero et al., 2003 [95]	France	Oscc	58	5.17%	16	PCR
Ostwald et al., 2003 [96]	Germany	Oscc	118	43.2%	6, 11, 16, 18	PCR/SB
Correnti et al., 2004 [97]	Venezuela	Oscc	16	50%	HR	PCR
Dahlgren et al., 2004 [98]	Sweden	Oscc	110	10.9%	16	PCR
Smith et al., 2004 [99]	USA	Oscc	106	9.4%	16, 33	PCR
Zhang et al., 2004 [100]	Japan	Oscc	73	34%	16, 18	PCR
Yang et al., 2004 [101]	Taiwan	Oscc	37	10.8%	16, 18	PCR
Koppikar et al., 2005 [102]	India	Oscc	101	31.7%	16, 18	PCR
Lo Muzio et al., 2005 [103]	Italy	Oscc	18	50%	n.d.	PCR
Boy et al., 2006 [104]	South Africa	Oscc	59	11%	18	PCR; ISH
El-Mofty and Patil 2006 [105]	USA	Oscc	94	30%	16, 31, 33	PCR
Nemes et al., 2006 [106]	Hungary	Oscc	79	41.7%	16	PCR
Rivero and Nunes 2006 [107]	Brazil	Oscc	23	0%	n.d.	PCR
Campisi et al., 2006 [108]	Italy	Oscc	63	38.1%	n.d	PCR
Furrer et al., 2006 [109]	Argentina	Oscc	14	42.8%	16, 18	PCR/SB
Koyama et al., 2007 [110]	Japan	Oscc	20	100%	18, 22, 16, 70	PCR
Sugiyama et al., 2007 [111]	Japan	Oscc	66	100%	16	PCR
Luo et al., 2007 [112]	Taiwan	Oscc	51	73%	—	PCR
Khovidhunkit et al., 2008 [113]	Thailand	Oscc	65	1.54%	16, 18	PCR
Szarka et al., 2009 [114]	Hungary	Oscc	65	47.7%	16, 18	PCR
Zhao et al., 2009 [115]	China	Oscc	52	40.4%	16, 18, 11, 6	PCR
Attner et al., 2010 [116]	Sweden	Oscc	87	68 (78)%	16, 33	PCR
Elango et al., 2011 [117]	India	Oscc	60	48%	16	PCR/IHC
Kristoffersen et al., 2012 [118]	Norway	Oscc	50	30%	6, 11, 16	PCR
Chen et al., 2012 [119]	Taiwan	Oscc	64	39%	16	ISH/IHC
Lee et al., 2012 [120]	Taiwan	Oscc	173	22%	16, 18	PCR

PCR: polymerase chain reaction; SB: southern blotting; ISH: *in situ* hybridization; IHC: immunohistochemistry.
